# Pharmacological Inhibition of Soluble Epoxide Hydrolase Ameliorates Diet-Induced Metabolic Syndrome in Rats

**DOI:** 10.1155/2012/758614

**Published:** 2011-10-10

**Authors:** Abishek Iyer, Kathleen Kauter, Md. Ashraful Alam, Sung Hee Hwang, Christophe Morisseau, Bruce D. Hammock, Lindsay Brown

**Affiliations:** ^1^School of Biomedical Sciences, The University of Queensland, Brisbane, QLD 4072, Australia; ^2^Department of Biological and Physical Sciences, University of Southern Queensland, Toowoomba, QLD 4350, Australia; ^3^Department of Entomology and University of California Davis Cancer Center, University of California, Davis, CA 95616, USA

## Abstract

The signs of metabolic syndrome following chronic excessive macronutrient intake include body weight gain, excess visceral adipose deposition, hyperglycaemia, glucose and insulin intolerances, hypertension, dyslipidaemia, endothelial damage, cardiovascular hypertrophy, inflammation, ventricular contractile dysfunction, fibrosis, and fatty liver disease. Recent studies show increased activity of soluble epoxide hydrolase (sEH) during obesity and metabolic dysfunction. We have tested whether sEH inhibition has therapeutic potential in a rat model of diet-induced metabolic syndrome. In these high-carbohydrate, high-fat-fed rats, chronic oral treatment with *trans*-4-[4-(3-adamantan-1-ylureido)-cyclohexyloxy]-benzoic acid (*t*-AUCB), a potent sEH inhibitor, alleviated the signs of metabolic syndrome *in vivo* including glucose, insulin, and lipid abnormalities, changes in pancreatic structure, increased systolic blood pressure, cardiovascular structural and functional abnormalities, and structural and functional changes in the liver. The present study describes the pharmacological responses to this selective sEH inhibitor in rats with the signs of diet-induced metabolic syndrome.

## 1. Introduction

Chronic metabolic dysfunction leading to obesity and diabetes represents a major challenge to health worldwide [1, 2]. Lifestyle changes such as an excessive caloric intake are indicated as important factors in initiating obesity and the associated metabolic and cardiovascular disorders [1–4]. This prevalent human condition is now collectively referred to as “metabolic syndrome” [3, 4]. Leading definitions of metabolic syndrome differ mainly in threshold values but in general emphasise the clustering of abdominal obesity, dyslipidaemia, hyperglycaemia, a prothrombotic state, and hypertension in increasing an individual's risk of developing type II diabetes mellitus, insulin resistance, and cardiovascular disease earlier in adult life [5–10]. This syndrome is associated with complications throughout the body such as excessive visceral fat deposition, hypertension, endothelial damage, cardiovascular hypertrophy, inflammation, atherosclerosis, ventricular contractile dysfunction, fibrosis and fatty liver disease [5, 8]. Identifying adequate therapeutic and preventive alternatives for this multifactorial syndrome has so far been challenging.

Arachidonic acid is the precursor of the eicosanoid family of signalling lipid mediators that modulate immune and inflammatory responses in the body [1, 11]. They are metabolised by cyclooxygenases, lipoxygenases, and cytochrome P450 monooxygenases to form biologically active products, including prostaglandins, leukotrienes, epoxyeicosatrienoic acids (EETs), and hydroxyeicosatetraenoic acids [1, 12, 13]. Pharmacological manipulation of the cyclooxygenase and lipoxygenase pathways improves adiposity and insulin sensitivity in humans and animal models [1, 14–16]. The cytochrome P450 enzymes generate EETs by catalysing the epoxidation of arachidonic acid [17]. EETs are endothelium-derived hyperpolarising factors that protect cells from ischaemic injury and possess anti-inflammatory responses in canine and rodent disease models [17]. These endogenous lipid mediators are converted into inactive diols by soluble epoxide hydrolase (sEH), and so inhibiting this enzyme would be expected to enhance the stability and therapeutic actions of EETs [17]. Orally active and selective sEH inhibitors that are based on 1,3-disubstituted urea and to a lesser extent amides and carbamates have been developed for chronic *in vivo* studies; *in vivo* these derivatives have proven to be antihypertensive and anti-inflammatory and protect the brain, heart, and kidney from damage [17]. Further, these derivatives produced analgesic responses in pain models [18]. 

**Figure 1:**
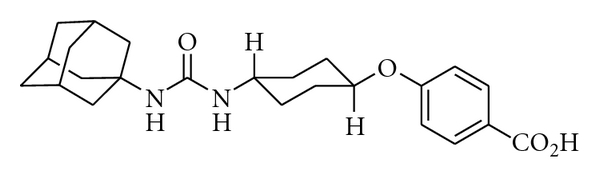
Chemical structure of the potent sEH inhibitor,* trans*-4-[4-(3-adamantan-1-ylureido)-cyclohexyloxy]-benzoic acid (*t*-AUCB).

Although it is well established that sEH inhibition improves cardiovascular and renal diseases [19–21], the therapeutic potential of sEH inhibition in diet-induced metabolic syndrome is still largely unknown [17]. To test the hypothesis that EETs are important in metabolic control, this study has evaluated *trans*-4-[4-(3-adamantan-1-ylureido)-cyclohexyloxy]-benzoic acid (*t*-AUCB) (Figure 1), a potent sEH inhibitor [22] with greater *in vivo* metabolic stability than many other sEH inhibitors [22], in a rat model of diet-induced metabolic syndrome. We have investigated whether chronic oral treatment with *t*-AUCB improved adiposity, control of blood glucose, insulin, and lipid homeostasis and the associated structural and functional changes in metabolic, liver, and cardiovascular systems induced by high-carbohydrate, high-fat feeding in rats.

## 2. Materials and Methods

### 2.1. Diet-Induced Metabolic Syndrome in Rats

All experimental protocols were approved by the University of Queensland Animal Experimentation Ethics Committee, under the guidelines of the National Health and Medical Research Council of Australia. Male Wistar rats (8-9 weeks old) were obtained from The University of Queensland Biological Resources facility. The rats were randomly divided into four experimental groups and were fed with either corn starch diet (CS; *n* = 24) or high-carbohydrate high-fat diet (HCHF; *n* = 24) for 16 weeks. The CS diet contained 570 g corn starch, 155 g powdered rat food (Specialty feeds, Glen Forest, WA, Australia), 25 g Hubble, Mendel, and Wakeman (HMW) salt mixture, and 250 g water per kilogram of food. HCHF diet contained 175 g fructose, 395 g sweetened condensed milk, 200 g beef tallow, 155 g powdered rat food, 25 g HMW salt mixture, and 50 g water per kilogram of food [23]. In addition, the drinking water for the HCHF group was supplemented with 25% fructose [23]. 12 rats from each CS and HCHF group were randomised into *t*-AUCB treatment at 8 weeks. Rats were given *ad libitum *access to food and water and were individually housed in a temperature-controlled 12 hours light-dark conditions. *t-*AUCB was administered in the drinking water in both CS and HCHF groups with the final dose calculated from daily drinking water consumption. Final doses of *t*-AUCB were 1.4 mg/Kg/day in CS-fed and 0.95 mg/Kg/day in HCHF-fed rats. *t*-AUCB was dissolved in 10 mL of distilled water made slightly basic with sodium hydroxide and then diluted to 1 L with distilled water.

### 2.2. Physiological Parameters

Body weight and food and water intakes were measured daily. Oral glucose tolerance and clearance tests have been previously described [23, 24]. Plasma lipids and enzyme concentrations were measured by the Veterinary Pathology Service of The University of Queensland, QLD, Australia [23, 24]. Systolic blood pressure of rats was measured as previously described [23–26].

### 2.3. Body Composition Measurements

Dual-energy X-ray absorptiometric (DXA) measurements were performed on the rats after 16 weeks of feeding (2 days before rats were euthanised for pathophysiological assessments) as previously described [23].

### 2.4. Experimental Protocol

Eight rats from each group were used for isolated Langendorff's preparations and vascular reactivity studies, and four rats per group were taken exclusively for histopathological analysis. For terminal experiments, rats were euthanised with Lethabarb (pentobarbitone sodium, 100 mg/kg, i.p.; Virbac, Peakhurst, NSW, Australia). After euthanasia, heparin (200 IU; Sigma-Aldrich Australia, Sydney, NSW, Australia) was injected through the right femoral vein. The abdomen was then opened, and blood (~6 mL) was withdrawn from the abdominal aorta and collected into heparinised tubes and centrifuged at 5,000 ×g for 15 minutes to obtain plasma. Plasma was stored at −20°C for further characterisation. Hearts were removed from rats for isolated Langendorff's preparation, and thoracic aortic rings were used for vascular reactivity studies as described below [25]. Liver, kidneys, and fat pads were removed from these rats and weighed. After perfusion studies, right ventricles from the hearts were removed and weighed whereas the left ventricle was weighed with septum. Weights of these organs were normalised relative to the tibial length at the time of removal (expressed as tissue weight in mg/mm tibial length) [23]. Heart, liver, kidney, and pancreas from the rats used for histopathological analysis were removed and fixed in appropriate solutions [23]. 

### 2.5. Structural Changes in Liver, Pancreas, and Adipose Tissue

Liver and pancreas were fixed with 10% neutral buffered formalin for three days. These tissue samples were dehydrated and then embedded in paraffin wax. Thin sections (5 *μ*m) of these tissues were cut and stained with Wright's staining for determination of inflammatory cell infiltration and for determining the fat vacuoles in liver. Liver sections were also stained with Milligan's stain to determine collagen deposition and to determine fat droplets. Routine histological stains used were Wright's for general histology and Milligan's for assessment of collagen deposition. Milligan's stained slides were visualised using a Nikon Eclipse 50*i *microscope (Kanagawa, Japan) fitted with a DSF*i*1 camera, and images were captured directly digitally (magnification ×200). Pancreatic sections were stained with aldehyde fuchsin staining following pretreatment with potassium permanganate (0.5%) and haematoxylin and eosin staining to determine infiltration of inflammatory cells. After staining, tissues were mounted, and pictures were taken with a Zeiss Microscope (magnification ×40 for haematoxylin and eosin and ×20 for aldehyde fuchsin, oil red “O”, and Milligan's stain). Islet area was quantified using NIH image J software (National Institute of Health, USA). Results are presented as mean ± SEM of the area of view. *α* and *β* cells in 12 islets were counted in each rat using NIH-image J software. Numbers of these cells are presented as mean ± SEM per islet [23]. Adipose tissue was fixed with 10% neutral buffered formalin for 1 day. Cryostat sections were prepared of this tissue in OCT as per normal histological procedures. Thick sections (40 *μ*m) of these tissues were cut and stained with Wright's staining for determination of inflammatory cell infiltration.

### 2.6. Cardiovascular Structure and Function

Echocardiographic examination was performed as previously described in all rats after 16 weeks [23, 27]. The isolated Langendorff's heart preparation was used to assess left ventricular function of the rats in all the groups as in previous studies [23, 25]. End diastolic pressures were obtained from 0 mmHg up to 30 mmHg for the calculation of diastolic stiffness constant (*κ*, dimensionless) as described in previous studies [23, 24, 26]. Thoracic aortic rings (4 mm in length) were suspended in an organ bath filled with Tyrode's physiological salt solution bubbled with 95% O_2_–5% CO_2_, maintained at 35°C, and allowed to stabilise at a resting tension of 10 mN. Cumulative concentration-response curves (contraction or relaxation) were obtained as in previous studies [25, 26]. Hearts were processed by two different procedures for histopathological studies as in previous studies [23, 25, 26].

### 2.7. Statistical Analysis

All data are presented as mean ± SEM. Differences between the groups were determined by one-way analysis of variance. Statistically significant variables were treated with the Neuman-Keuls *post hoc *test to compare all the groups of animals. For body weight data, Student's *t*-tests were performed. All statistical analyses were performed using Graph Pad Prism version 5 for Windows (San Diego, Calif, USA) with a *P* value of <0.05 considered as statistically significant.

## 3. Results

### 3.1. HCHF Diet Induces Signs of the Metabolic Syndrome

Young male Wistar rats fed HCHF diet showed progressive increases in body weight, abdominal fat deposition, and whole body fat mass along with impaired glucose tolerance, plasma lipid abnormalities, hyperinsulinaemia, and increased plasma leptin concentrations [23]. Cardiovascular changes included increased systolic blood pressure and endothelial dysfunction together with inflammation, fibrosis, hypertrophy, and increased stiffness of the left ventricle of the heart [23]. The liver showed increased wet weight, fat deposition, inflammation, and fibrosis with increased plasma activity of liver enzymes. The pancreas showed increased islet size. Treatment with *t*-AUCB from week 8 to 16 attenuated the metabolic, liver, and cardiovascular abnormalities, outlined below, but did not affect the increased body weight, abdominal fat deposition, and whole body fat mass (Table 1).

### 3.2. Treatment with *t*-AUCB Attenuated Metabolic Abnormalities

**Figure 2:**
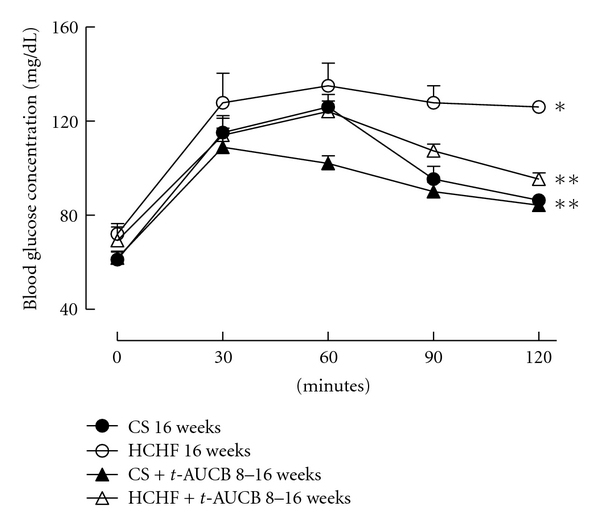
Plasma glucose concentrations following oral gavage of glucose (2 g/kg) recorded after 16 weeks for rats fed with corn starch (CS), high carbohydrate high fat (HCHF), corn starch with *t*-AUCB (CS + *t*-AUCB), and high carbohydrate high fat with *t*-AUCB (HCHF + *t*-AUCB). *P < 0.05 versus CS-fed rats; **P < 0.05 versus HCHF-fed rats.

**Figure 3:**
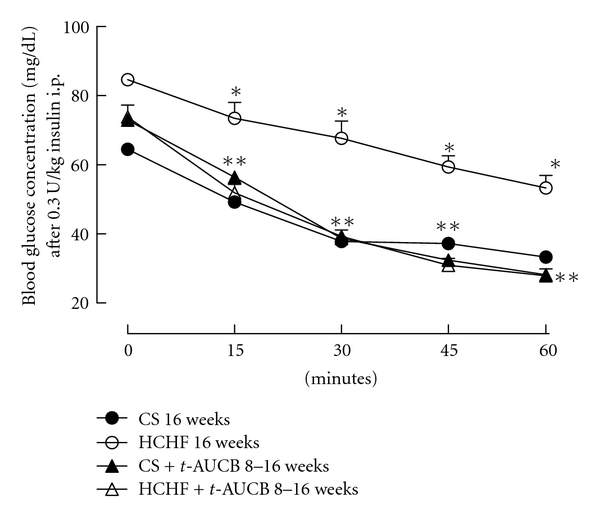
Plasma glucose concentrations following 0.3 U/Kg insulin i.p. dose after 16 weeks for rats fed with corn starch (CS), high carbohydrate high fat (HCHF), corn starch with *t*-AUCB (CS + *t*-AUCB), and high carbohydrate high fat with *t*-AUCB (HCHF + *t*-AUCB). *P < 0.05 versus CS-fed rats; **P < 0.05 versus HCHF-fed rats.

**Figure 4:**
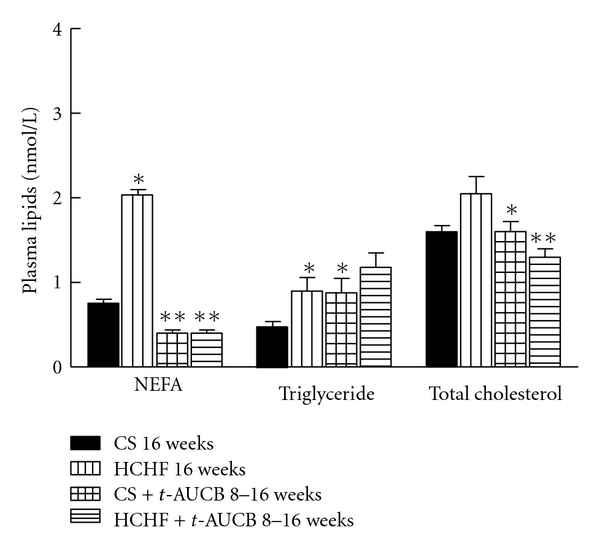
Plasma lipid concentrations after 16 weeks for rats fed with corn starch (CS), high carbohydrate high fat (HCHF), corn starch with *t*-AUCB (CS + *t*-AUCB), and high carbohydrate high fat with *t*-AUCB (HCHF + *t*-AUCB). *P < 0.05 versus CS-fed rats; **P < 0.05 versus HCHF-fed rats.

Overnight fasting blood glucose concentrations did not differ among the experimental groups. Following oral glucose loading, blood glucose concentrations in CS-fed rats increased after 30 mins then decreased rapidly and returned to fasting glucose concentrations at 120 mins after glucose loading (Figure 2). In HCHF-fed rats, blood glucose concentrations decreased much more slowly and were higher at 120 mins after glucose loading compared to CS-fed rats, suggesting glucose intolerance (Figure 2). Blood glucose concentrations at both 4-hour fasting and 75 mins after intraperitoneal administration of insulin were much higher in HCHF-fed rats compared to CS-fed rats suggesting insulin intolerance (Figure 3). Both of the above features were attenuated with *t*-AUCB treatment in HCHF-fed rats (Figures 2 and 3). Plasma concentrations of insulin were increased with HCHF diet after 16 weeks in comparison to CS diet (Table 1). Plasma leptin concentrations did not increase with the HCHF diet compared to CS diet at 16 weeks (Table 1). *t-*AUCB did not prevent the increased insulin concentrations in HCHF-fed rats but further increased insulin concentrations in CS-treated rats (Table 1). Further, plasma concentrations of NEFA, total cholesterol, and triglycerides were elevated with HCHF feeding compared to CS-fed controls (Figure 4). Treatment with *t*-AUCB completely normalised these increased NEFA and total cholesterol concentrations in HCHF-fed rats (Figure 4). In CS-fed rats treated with *t*-AUCB, there was a further reduction in NEFA concentrations compared to control rats (Figure 4). There was no change in increased plasma triglycerides with *t*-AUCB treatment in HCHF-fed rats (Figure 4).

### 3.3. Structure and Function of Liver, Pancreas, and Adipose Tissue Are Moderated by *t*-AUCB

**Figure 5:**
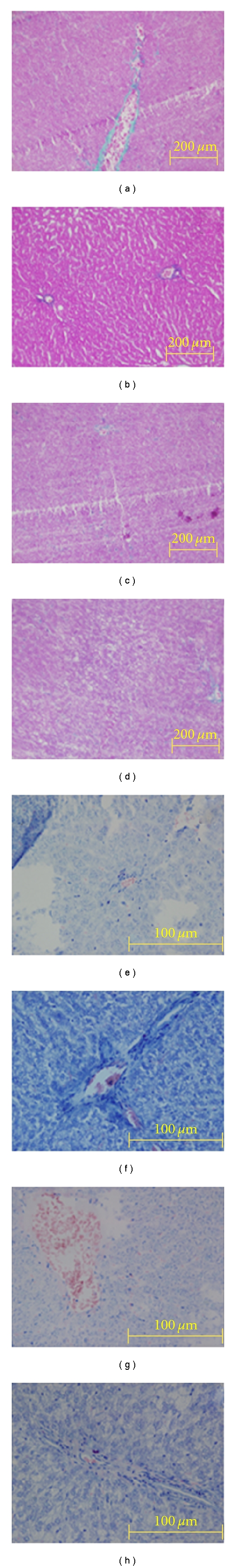
Structural changes and mild inflammation in the liver after 16 weeks for rats fed with corn starch (CS) (a) and (e), high carbohydrate high fat (HCHF) (b) and (f), corn starch with *t*-AUCB (CS + *t*-AUCB) (c) and (g), and high carbohydrate high fat with *t*-AUCB (HCHF + *t*-AUCB) (d) and (h).

Postmortem liver weights (normalised to tibial length) from HCHF-fed rats were increased compared to CS-fed rats (Table 1). Treatment with *t*-AUCB did not prevent this organ's hypertrophy induced by HCHF feeding (Table 1). Histological analysis of liver samples showed HCHF diet-fed rats to have increased infiltration of inflammatory cells and increased deposition of collagen around the blood vessels compared to CS diet-fed rats (Figure 5). HCHF diet-fed rats showed deposition of fat droplets in liver, which were rarely observed in livers from CS diet-fed rats (Figure 5). Also, the size of fat vacuoles was larger in HCHF diet-fed rats (Figure 5). *t*-AUCB treatment attenuated the mild steatosis and increased hypertrophy and vacuole size seen in HCHF-fed rats without affecting the increased inflammatory cell infiltration (Figure 5). HCHF diet resulted in increased plasma activity of AST, ALT, ALP, and LDH compared to CS diet-fed rats indicating liver cell damage (Table 1). Treatment with *t*-AUCB attenuated the increased AST and LDH concentrations but not the ALT and ALP concentrations (Table 1). 

**Figure 6:**
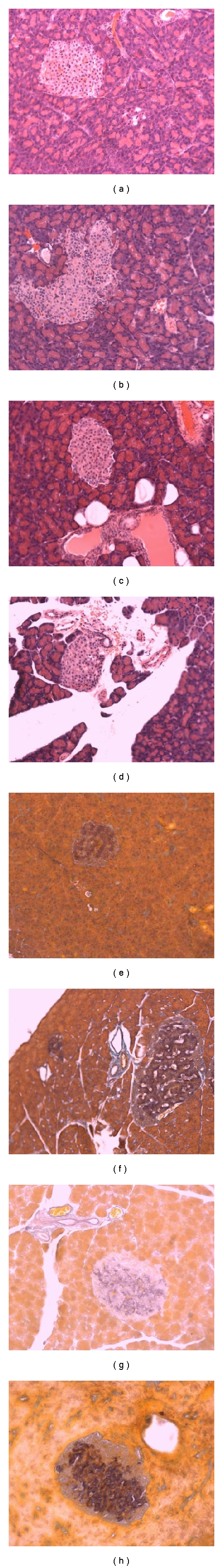
Structural changes in the pancreatic islets of Langerhans after 16 weeks for rats fed with corn starch (CS) (a) and (e), high carbohydrate high fat (HCHF) (b) and (f), corn starch with *t*-AUCB (CS + *t*-AUCB) (c) and (g), and high carbohydrate high fat with *t*-AUCB (HCHF) (d), (h), Magnification 20x.

**Figure 7:**
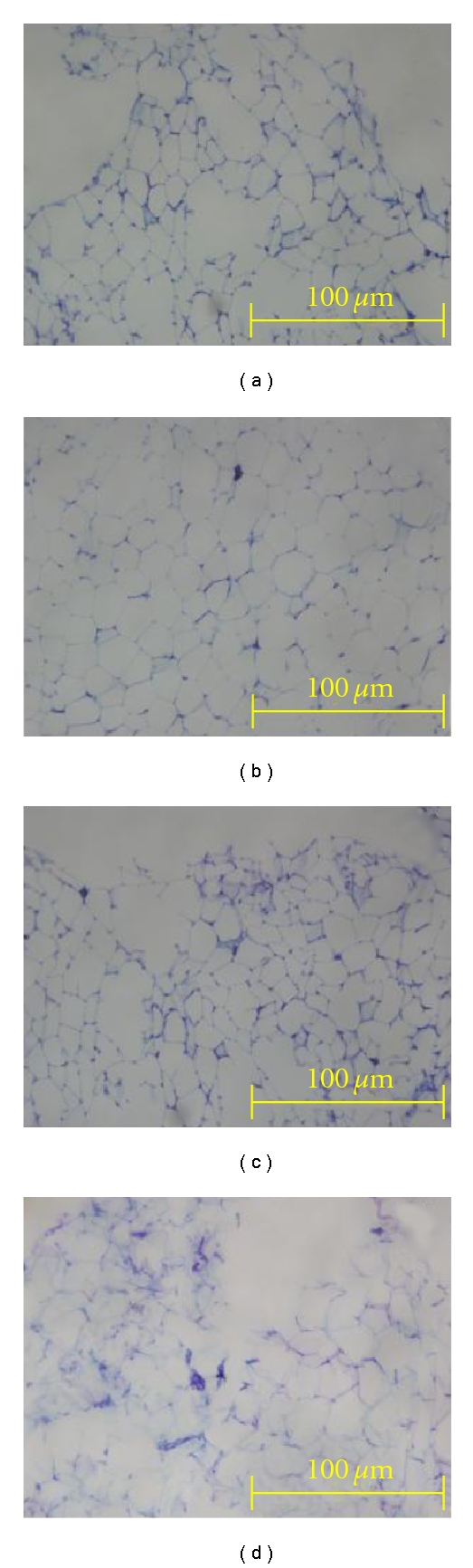
Structural changes and mild inflammation in the adipose tissue after 16 weeks for rats fed with corn starch (CS) (a), high carbohydrate high fat (HCHF) (b), corn starch with *t*-AUCB (CS + *t*-AUCB) (c), and high carbohydrate high fat with *t*-AUCB (HCHF + *t*-AUCB) (d).

The pancreas from HCHF diet-fed rats showed an increased number of inflammatory cells (Figure 5) with increased size of islets of Langerhans compared with CS diet-fed rats. Numbers of both *α* and *β* cells were also increased in HCHF diet-fed rats compared to CS diet-fed rats. Treatment with *t*-AUCB did not change the islets size in CS- or in HCHF-fed rats. *t*-AUCB treatment increased the number of both *α* and *β* cells in CS diet-fed rats but only increased *β*-cell numbers in HCHF-fed rats (Figure 6). Histological analysis of the adipose tissue samples showed increased infiltration of immune inflammatory cells with HCHF feeding at 16 weeks compared to CS-fed rats (Figure 7). Treatment with *t*-AUCB did not reduce this increased infiltration (Figure 7).

### 3.4. Treatment with *t*-AUCB Attenuated Changes in Cardiovascular Structure and Function

**Figure 8:**
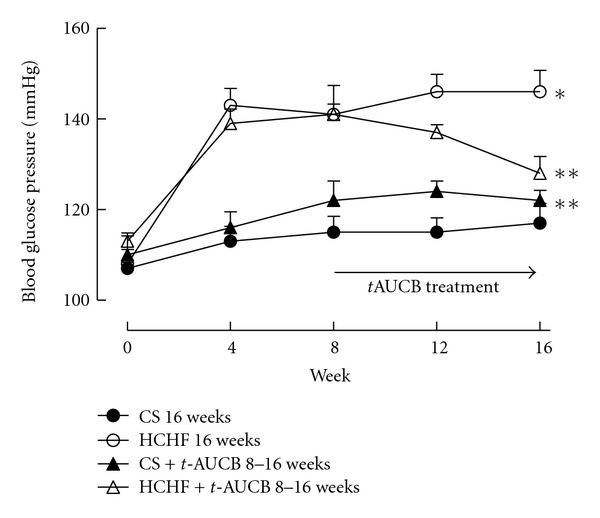
Systolic blood pressure measurements for rats fed with corn starch (CS), high carbohydrate high fat (HCHF), corn starch with *t*-AUCB (CS + *t*-AUCB), and high carbohydrate high fat with *t*-AUCB (HCHF + *t*-AUCB). *P < 0.05 versus CS-fed rats; **P < 0.05 versus HCHF-fed rats.

Heart rate did not vary among the experimental groups (Table 1). Systolic blood pressure in HCHF-fed rats increased sharply at week 4 and remained elevated until the end of the study protocol compared to CS-fed rats (Figure 8). Treatment with *t*-AUCB completely reversed the increased systolic blood pressure in HCHF-fed rats at 16 weeks (Figure 8). Echocardiographic assessment of HCHF-fed rats showed ventricular dilatation (increased left ventricular end diastolic dimensions), increased systolic volume, and increased estimated left ventricular mass (Table 1). Treatment with *t*-AUCB from week 8 to week 16 attenuated all these structural changes in HCHF-fed rats (Table 1). 

**Figure 9:**
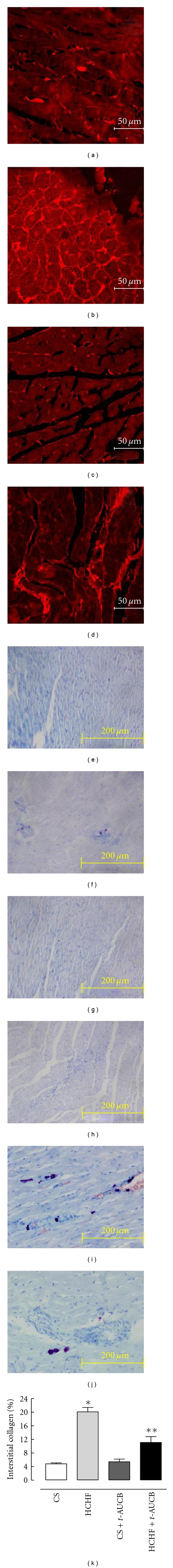
Inflammation and fibrosis in the heart. Representative images of left ventricular interstitial collagen deposition stained in picrosirius red staining (×40) after 16 weeks in CS (a), HCHF (b), CS + *t*-AUCB (c), HCHF + *t*-AUCB (d). Wright's staining of LV of the heart (×40) showing infiltration of inflammatory cells after 16 weeks in CS (e), HCHF (f), CS + *t*-AUCB (g), HCHF + *t*-AUCB (h). Wright's staining of LV of the heart (×100) showing infiltration of mast cells after 16 weeks in HCHF (i), HCHF + *t*-AUCB (j). Summary data of left ventricular interstitial collagen deposition (k) after 16 weeks in CS, HCHF, CS + *t*-AUCB, HCHF + *t*-AUCB. *P < 0.05 versus CS-fed rats, **P < 0.05 versus HCHF-fed rats.

Many inflammatory cells were observed in the left ventricle of HCHF-fed rats, whereas the number of inflammatory cells in the left ventricle of CS-fed rats was very low (Figure 9). Treatment with *t*-AUCB, in general, did not prevent this increased infiltration of inflammatory cells but selectively prevented increased mast cells into the left ventricle in HCHF-fed rats. Interstitial collagen contents in left ventricle were increased in HCHF-fed rats compared with CS-fed rats (Figure 9). Treatment with *t*-AUCB attenuated this increase in interstitial collagen deposition in HCHF-fed rats (Figure 9). The isolated Langendorff's heart preparation showed increased diastolic stiffness in HCHF-fed rats at 16 weeks compared to CS-fed rats (Table 1). Treatment with *t-*AUCB from week 8 to week 16 completely reversed this increase in left ventricular diastolic stiffness in HCHF-fed rats (Table 1).

**Figure 10:**
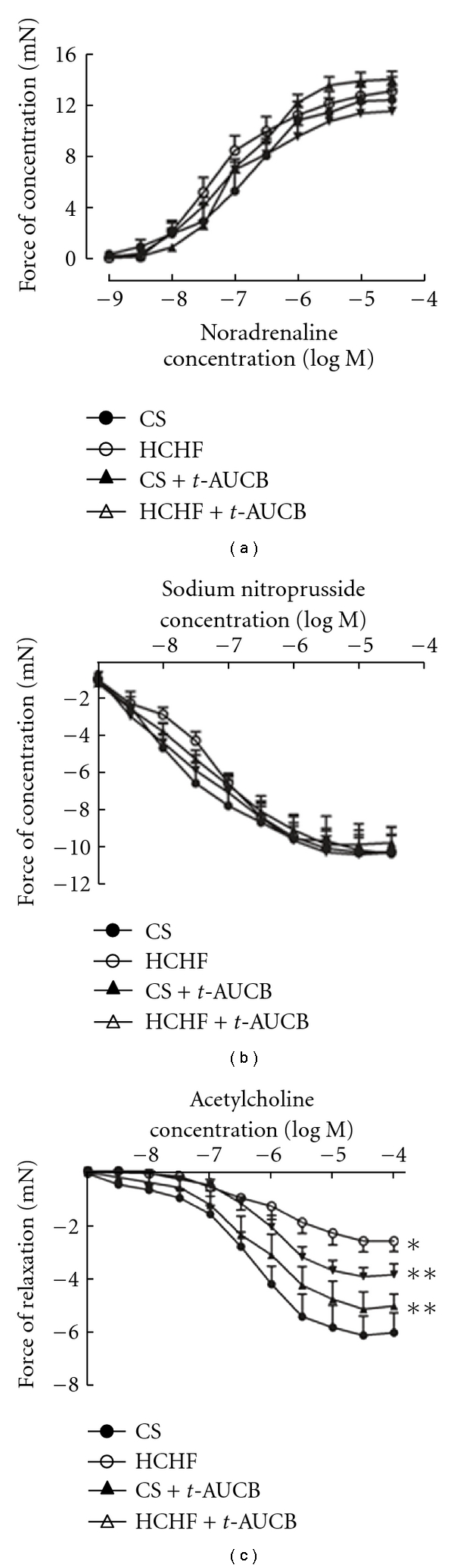
Cumulative concentration-response curves for noradrenaline (a), sodium nitroprusside (b), and acetylcholine (c) in thoracic aortic rings after 16 weeks from CS-, HCHF-, CS + *t*-AUCB-, HCHF + *t*-AUCB-treated rats. *P < 0.05 versus CS-fed rats; **P < 0.05 versus HCHF-fed rats.

Lastly, organ bath studies showed no change in vascular responses to noradrenaline (constriction) and sodium nitroprusside (relaxation) among all treatment groups (Figure 10). In contrast, HCHF-fed rats showed pronounced endothelial dysfunction, seen as reduced vascular relaxation responses to acetylcholine compared to both CS-fed rats (Figure 10). Treatment with *t*-AUCB attenuated this decreased response to acetylcholine (Figure 10).

## 4. Discussion

The signs of metabolic syndrome following chronic excessive macronutrient intake include body weight gain, excess visceral adipose deposition, hyperglycaemia, glucose and insulin intolerances, hypertension, dyslipidaemia, endothelial damage, cardiovascular hypertrophy, inflammation, ventricular contractile dysfunction, fibrosis, and fatty liver disease. Although it is well recognised that the inhibition of sEH lowers blood pressure in different rat models [19, 21, 28], the therapeutic potential of sEH inhibition in the control of diet-induced adiposity, metabolic and cardiovascular dysfunction are unknown. In this study, we show that sEH inhibition has therapeutic potential in the control of diet-induced prediabetes and metabolic syndrome. We report that chronic oral treatment with *t*-AUCB (~1 mg/kg/day), a potent sEH inhibitor [22], alleviated the symptoms of metabolic syndrome *in vivo* including glucose, insulin and lipid abnormalities, changes in pancreatic structure, increased systolic blood pressure, and cardiovascular and liver structural and functional abnormalities induced by chronic high-carbohydrate high-fat feeding in rats.

Clinical, animal and *in vitro* studies support links between obesity, insulin resistance, type II diabetes, metabolic dysfunction, and inflammation. Anti-inflammatory agents such as NSAIDs, salicylates, and aspirin reduce the severity of metabolic dysfunction [1, 2]. Local adipose tissue inflammation and inflammatory lipid mediators including EETs have been suggested to play important roles in regulating adipocyte function and metabolic homeostasis [1]. A recent study reported that sEH mRNA and protein concentrations in adipose tissue did not differ between normal- and fat-fed animals, but total adipose sEH activity was increased in obese mice, with a large increase during maturation of adipocytes [29]. Given the involvement of sEH in inflammation and also the increased activity of sEH during obesity and metabolic dysfunction, we hypothesised that increasing EET concentrations by sEH inhibition may be important in controlling obesity and the symptoms of the metabolic syndrome. Chronic oral treatment with *t-*AUCB improved metabolic and cardiovascular symptoms but did not alter body weight gain, excess visceral adipose deposition, or infiltration of immune inflammatory cells into the adipose tissue compared to untreated rats. This implies that *t*-AUCB acts to increase the anti-inflammatory EETs as metabolites of arachidonate produced by phospholipase A2 from inflammatory cells, rather than preventing infiltration of these immune inflammatory cells. It is well accepted that inhibition of sEH increases EET concentrations by decreasing their conversion into inactive diols (DHETs) [20]. Further, *t*-AUCB treatment reduces the production of DHETs and increases the ratios of EETs to DHETs in the plasma of lipopolysaccharide-treated mice [22]. Similarly, sEH knock-out mice have higher plasma ratios of EETs to DHETs than wild-type mice [30]. Thus, it is likely that sEH inhibition by *t*-AUCB may prevent adipocyte dysfunction downstream of immune inflammatory cell infiltration without affecting adiposity [1].

Further, changes in glucose, insulin, and lipid homeostasis are characteristic of insulin resistance, type II diabetes, and metabolic syndrome. There is growing incidental evidence to suggest the involvement of sEH in glucose, insulin, and lipid abnormalities. CYP2J protein, which generates EETs, is expressed in human and rat pancreatic tissues where significant amounts of endogenous EETs have been detected [31]. Also, EETs are potent mediators of insulin release in isolated rat islets [32]. In this study, we investigated whether *t*-AUCB treatment improves glucose and insulin intolerances and elevated plasma lipids induced by high-carbohydrate high-fat feeding. Oral glucose and intraperitoneal insulin tolerance tests showed improved tolerance to both glucose and insulin in rats treated with *t*-AUCB compared to untreated ones. A recent study assessed the role of sEH in glucose and insulin homeostasis in streptozotocin- (STZ-) treated mice using both sEH knockout and sEH inhibition using *t*-AUCB [33]. This study showed that both sEH knockout and *t*-AUCB treatment prevented hyperglycaemia in type I diabetes through enhanced islet glucose-stimulated insulin release by the alternate pathway and decreased islet cell apoptosis [33]. Further, a recent study supports our results and hypothesis by showing that inhibition of sEH restored glucose homeostasis and insulin signalling together with increased pancreatic islet size in a mice model of type II diabetes [34]. These could be the same mechanisms through which *t*-AUCB improved glucose and insulin abnormalities in our study.

In patients with type II diabetes mellitus, single nucleotide polymorphisms in the sEH gene were associated with an increased risk of cardiovascular disease [35] and hypertension [36]. Although it is well established that sEH inhibitors have beneficial effects in cardiovascular diseases [19–21], their therapeutic potential in metabolic syndrome-induced cardiovascular changes is unknown. In the present study, *t*-AUCB treatment attenuated the cardiovascular changes induced by high-carbohydrate high-fat feeding including elevated systolic blood pressure and endothelial dysfunction fibrosis, hypertrophy, and increased stiffness in the left ventricle of the heart. Treatment with *t*-AUCB also showed improved plasma liver enzymes and decreased steatosis compared to untreated animals.

The sEH inhibitors show target engagement in many studies in terms of increasing the plasma concentrations of epoxy fatty acids and decreasing the concentrations of the corresponding diols [37]. The broad and usually beneficial biological activities observed with the sEH inhibitors may occur from several causes. One is that not only the 4 regioisomers of EETs are being stabilised but also the epoxides of other fatty acids are being stabilised including linoleate, eicosapentaenoic acid, and docosahexaenoic acid [38], but the concentrations of the sometimes inflammatory diols are being reduced [39]. Observing multiple biological effects following the administration of a drug that influences the arachidonate cascade is common in pharmacology with the nonsteroidal inflammatory drugs being a case in point. Although a receptor is not known for EETs, they are known to block nuclear translocation of NF*κ*B [40] and to downregulate induced iNOS and COX 2 [41], all of which influence many downstream biological activities. Further, the current paradigm in the origin of metabolic syndrome is that adipocyte dysfunction promotes the metabolic and cardiovascular symptoms of the metabolic syndrome [1, 5]. sEH activity in adipose tissue increases with adiposity and correlates with the associated metabolic dysfunction in obesity [29]. Thus, attenuating adiposity dysfunction by inhibiting the increased sEH activity in adipose tissue using *t*-AUCB may account for the beneficial responses seen in all the other metabolic systems.

The sEH inhibitor, *t*-AUCB, like any pharmacological probe could be producing off-target effects. However, the low nanomolar inhibitory concentrations and picomolar Ki of *t*-AUCB argue for selectivity. This class of compounds has shown few off-target effects with most of these being in the micromolar or nanomolar range. The same biological responses have been observed in multiple systems using radically different sEH inhibitor structures. Although not used in this study, the EET antagonist, 14,15-EEZE, reversed the responses to sEH inhibitors in other models while EET administration enhanced the responses. Some of these responses were also mimicked in rodent models of diabetes using genetic knockouts of the sEH [34]. 

In summary, the present study provides new information about the therapeutic potential of a selective sEH inhibitor in treating the signs of diet-induced metabolic syndrome, as well as the beneficial responses of sEH inhibition on metabolic syndrome-induced cardiovascular and liver abnormalities. The great diversity of biological effects resulting from increased concentrations of EETs suggests the presence of multiple receptors with the assumption that at least some G-protein-coupled receptors are involved [1]. Further research into identifying the receptors that mediate EET responses in metabolically relevant tissues would throw light on the mechanisms of action for their therapeutic responses. sEH inhibitors also synergise the anti-inflammatory actions of NSAIDs, which suggests that low doses of NSAIDs could be used in combination to reduce the symptoms of metabolic syndrome and adiposity possibly without compromising innate immunity [1, 42]. Thus, targeting sEH with selective inhibitors either alone or in combination with low doses of NSAIDs could reduce metabolic and cardiovascular dysfunction in metabolic syndrome.

## Figures and Tables

**Table 1:** Physiological parameters of CS-, HCHF-, CS + *t*-AUCB-, HCHF + *t*-AUCB-treated rats. *P < 0.05 versus CS-fed rats; **P < 0.05 versus HCHF-fed rats.

Parameter	CS (16 weeks)	HCHF (16 weeks)	CS + *t*-AUCB (16 weeks)	HCHF + *t*-AUCB (16 weeks)
Body weight @ week 16 (g)	422 ± 6 (*n* = 6)	515.8 ± 11.9 (*n* = 6)	415 ± 6 (*n* = 6)	515.7 ± 13 (*n* = 6)
Abdominal fat deposition (mg/mm tibial length)	401 ± 56 (*n* = 6)	746 ± 67* (*n* = 6)	270 ± 38 (*n* = 6)	674 ± 99* (*n* = 6)
Total fat mass (g)	56 ± 5 (*n* = 6)	139.4 ± 16* (*n* = 6)	46.7 ± 8 (*n* = 6)	154.3 ± 19* (*n* = 6)
Heart rate (bpm)	249 ± 9 (*n* = 6)	251 ± 21 (*n* = 6)	262 ± 13 (*n* = 6)	265 ± 8 (*n* = 6)
LVIDd (mm)	6.73 ± 0.2 (*n* = 6)	7.54 ± 0.18* (*n* = 6)	6.8 ± 0.2** (*n* = 6)	6.8 ± 0.2** (*n* = 6)
LVPWd (mm)	1.69 ± 0.1 (*n* = 6)	1.95 ± 0.1 (*n* = 6)	1.69 ± 0.08 (*n* = 6)	1.9 ± 0.1 (*n* = 6)
LVIDs (mm)	3.9 ± 0.3 (*n* = 6)	4.9 ± 0.2* (*n* = 6)	3.6 ± 0.2** (*n* = 6)	4.2 ± 0.2** (*n* = 6)
E/A ratio	1.6 ± 0.1 (*n* = 6)	1.2 ± 0.1* (*n* = 6)	2.0 ± 0.2** (*n* = 6)	1.5 ± 0.04** (*n* = 6)
Ejection fraction (%)	79 ± 4 (*n* = 6)	72 ± 2 (*n* = 6)	85 ± 2** (*n* = 6)	76 ± 3 (*n* = 6)
Cardiac output (mL/min)	63.4 ± 6.4 (*n* = 6)	83.8 ± 9 (*n* = 6)	81.8 ± 10 (*n* = 6)	72.2 ± 7 (*n* = 6)
Estimated LV mass (g)	0.74 ± 0.05 (*n* = 6)	1.0 ± 0.05* (*n* = 6)	0.77 ± 0.05** (*n* = 6)	0.84 ± 0.04** (*n* = 6)
Diastolic stiffness constant (*κ*)	20.5 ± 1.9 (*n* = 6)	28.9 ± 1* (*n* = 6)	22.8 ± 0.4** (*n* = 6)	20.1 ± 0.1** (*n* = 6)
Plasma insulin concentrations	2.2 ± 0.4 (*n* = 5)	4.2 ± 0.3* (*n* = 5)	4.7 ± 1.1* (*n* = 5)	5.2 ± 1* (*n* = 5)
Plasma leptin concentrations	6.8 ± 0.86 (*n* = 5)	8.6 ± 1.1 (*n* = 5)	6.9 ± 0.8 (*n* = 5)	9.7 ± 0.5 (*n* = 5)
Plasma ALT activity, U/L	37 ± 0.6 (*n* = 5)	59.6 ± 0.3* (*n* = 5)	52.3 ± 2* (*n* = 5)	49.4 ± 3.2* (*n* = 5)
Plasma AST activity, U/L	73.2 ± 5.6 (*n* = 5)	105.3 ± 9* (*n* = 5)	68.2 ± 3.2** (*n* = 5)	69.7 ± 5.7** (*n* = 5)
Plasma ALP activity, U/L	174 ± 19 (*n* = 5)	251 ± 21* (*n* = 5)	192 ± 11** (*n* = 5)	274 ± 29* (*n* = 5)
Plasma LDH activity, U/L	204 ± 23 (*n* = 5)	497 ± 14* (*n* = 5)	180 ± 56** (*n* = 5)	184 ± 26** (*n* = 5)
LV + septum (mg/mm tibial length)	18.9 ± 0.9 (*n* = 6)	21.8 ± 1.1 (*n* = 6)	20.3 ± 1.5 (*n* = 6)	20.5 ± 0.8 (*n* = 6)
Right ventricle (mg/mm tibial length)	4.14 ± 0.3 (*n* = 6)	4.48 ± 0.3 (*n* = 6)	3.7 ± 0.2 (*n* = 6)	4.4 ± 0.3 (*n* = 6)
Liver (mg/mm tibial length)	234 ± 14 (*n* = 6)	287 ± 12* (*n* = 6)	264 ± 15 (*n* = 6)	292 ± 19* (*n* = 6)
Islets as % area of pancreas	6.2 ± 1.4 (*n* = 6)	15.3 ± 1.5 (*n* = 6)	5.2 ± 0.1 (*n* = 6)	16.7 ± 1.3 (*n* = 6)
Number of *α* cells/islet	16.9 ± 0.5 (*n* = 6)	39.3 ± 1.2 (*n* = 6)	22.4 ± 1.9 (*n* = 6)	38.6 ± 1.4 (*n* = 6)
Number of *β* cells/islet	72.3 ± 2.8 (*n* = 6)	124.7 ± 7.2 (*n* = 6)	117.1 ± 4.1 (*n* = 6)	149.8 ± 6.3 (*n* = 6)

## References

[1] Iyer A, Fairlie DP, Prins JB, Hammock BD, Brown L (2010). Inflammatory lipid mediators in adipocyte function and obesity. *Nature Reviews Endocrinology*.

[2] Iyer A, Brown L (2010). Lipid mediators and inflammation in glucose intolerance and insulin resistance. *Drug Discovery Today: Disease Mechanisms*.

[3] Potenza MV, Mechanick JI (2009). The metabolic syndrome: definition, global impact, and pathophysiology. *Nutrition in Clinical Practice*.

[4] Simmons RK, Alberti KG, Gale EA (2010). The metabolic syndrome: useful concept or clinical tool? Report of a WHO expert consultation. *Diabetologia*.

[5] van Gaal LF, Mertens IL, De Block CE (2006). Mechanisms linking obesity with cardiovascular disease. *Nature*.

[6] Reaven GM (1988). Role of insulin resistance in human disease. *Diabetes*.

[7] Dandona P, Aljada A, Chaudhuri A, Mohanty P, Garg R (2005). Metabolic syndrome: a comprehensive perspective based on interactions between obesity, diabetes, and inflammation. *Circulation*.

[8] Zimmet PZ, Alberti KG, Shaw JE (2005). Mainstreaming the metabolic syndrome: a definitive defenition. *Medical Journal of Australia*.

[9] Ferrannini E (2006). Is insulin resistance the cause of the metabolic syndrome?. *Annals of Medicine*.

[10] Symonds ME, Sebert SP, Hyatt MA, Budge H (2009). Nutritional programming of the metabolic syndrome. *Nature Reviews Endocrinology*.

[11] Habenicht AH, Salbach P, Goerig M (1990). The LDL receptor pathway delivers arachidonic acid for eicosanoid formation in cells stimulated by platelet-derived growth factor. *Nature*.

[12] Kudo I, Murakami M (2002). Phospholipase A2 enzymes. *Prostaglandins and Other Lipid Mediators*.

[13] Lambeau G, Gelb MH (2008). Biochemistry and physiology of mammalian secreted phospholipases A 2. *Annual Review of Biochemistry*.

[14] Shi X, Ding M, Dong Z (1999). Antioxidant properties of aspirin: characterization of the ability of aspirin to inhibit silica-induced lipid peroxidation, DNA damage, NF-*κ*B activation, and TNF-*α* production. *Molecular and Cellular Biochemistry*.

[15] Yuan M, Konstantopoulos N, Lee J (2001). Reversal of obesity- and diet-induced insulin resistance with salicylates or targeted disruption of Ikk*β*. *Science*.

[16] Bastard JP, Maachi M, Lagathu C (2006). Recent advances in the relationship between obesity, inflammation, and insulin resistance. *European Cytokine Network*.

[17] Imig JD, Hammock BD (2009). Soluble epoxide hydrolase as a therapeutic target for cardiovascular diseases. *Nature Reviews Drug Discovery*.

[18] Inceoglu B, Wagner K, Schebb NH (2011). Analgesia mediated by soluble epoxide hydrolase inhibitors is dependent on cAMP. *Proceedings of the National Academy of Sciences of the United States of America*.

[19] Imig JD, Zhao X, Zaharis CZ (2005). An orally active epoxide hydrolase inhibitor lowers blood pressure and provides renal protection in salt-sensitive hypertension. *Hypertension.*.

[20] Chiamvimonvat N, Ho CM, Tsai HJ, Hammock BD (2007). The soluble epoxide hydrolase as a pharmaceutical target for hypertension. *Journal of Cardiovascular Pharmacology*.

[21] Loch D, Hoey A, Morisseau C, Hammock BO, Brown L (2007). Prevention of hypertension in DOCA-salt rats by an inhibitor of soluble epoxide hydrolase. *Cell Biochemistry and Biophysics*.

[22] Liu JY, Tsai HJ, Hwang SH, Jones PD, Morisseau C, Hammock BD (2009). Pharmacokinetic optimization of four soluble epoxide hydrolase inhibitors for use in a murine model of inflammation. *British Journal of Pharmacology*.

[23] Panchal SK, Poudyal H, Iyer A (2011). High-carbohydrate, high-fat diet-induced metabolic syndrome and cardiovascular remodeling in rats. *Journal of Cardiovascular Pharmacology*.

[24] Iyer A, Brown L (2011). Fermented wheat germ extract (Avemar) in the treatment of cardiac remodeling and metabolic symptoms in rats. *Evidence-Based Complementary and Alternative Medicine*.

[25] Iyer A, Fenning A, Lim J (2010). Antifibrotic activity of an inhibitor of histone deacetylases in DOCA-salt hypertensive rats: research paper. *British Journal of Pharmacology*.

[26] Chan V, Fenning A, Iyer A, Hoey A, Brown L (2011). Resveratrol improves cardiovascular function in DOCA-salt hypertensive rats. *Current Pharmaceutical Biotechnology*.

[27] Litwin SE, Katz SE, Morgan JP, Douglas PS (1994). Serial echocardiographic assessment of left ventricular geometry and function after large myocardial infarction in the rat. *Circulation*.

[28] Imig JD, Zhao X, Capdevila JH, Morisseau C, Hammock BD (2002). Soluble epoxide hydrolase inhibition lowers arterial blood pressure in angiotensin II hypertension. *Hypertension*.

[29] de Taeye BM, Morisseau C, Coyle J (2010). Expression and regulation of soluble epoxide hydrolase in adipose tissue. *Obesity*.

[30] Seubert JM, Sinal CJ, Graves J (2006). Role of soluble epoxide hydrolase in postischemic recovery of heart contractile function. *Circulation Research*.

[31] Zeldin DC, Foley J, Boyle JE (1997). Predominant expression of an arachidonate epoxygenase in islets of langerhans cells in human and rat pancreas. *Endocrinology*.

[32] Falck JR, Manna S, Moltz J, Chacos N, Capdevila J (1983). Epoxyeicosatrienoic acids stimulate glucagon and insulin release from isolated rat pancreatic islets. *Biochemical and Biophysical Research Communications*.

[33] Luo P, Chang HH, Zhou Y (2010). Inhibition or deletion of soluble epoxide hydrolase prevents hyperglycemia, promotes insulin secretion, and reduces islet apoptosis. *Journal of Pharmacology and Experimental Therapeutics*.

[34] Luria A, Bettaieb A, Xi Y (2011). Soluble epoxide hydrolase deficiency alters pancreatic islet size and improves glucose homeostasis in a model of insulin resistance. *Proceedings of the National Academy of Sciences of the United States of America*.

[35] Lee CR, North KE, Bray MS (2006). Genetic variation in soluble epoxide hydrolase (EPHX2) and risk of coronary heart disease: the Atherosclerosis Risk in Communities (ARIC) study. *Human Molecular Genetics*.

[36] Burdon KP, Lehtinen AB, Langefeld CD (2008). Genetic analysis of the soluble epoxide hydrolase gene, EPHX2, in subclinical cardiovascular disease in the Diabetes Heart Study. *Diabetes and Vascular Disease Research*.

[37] Yang J, Schmelzer K, Georgi K, Hammock BD (2009). Quantitative profiling method for oxylipin metabolome by liquid chromatography electrospray ionization tandem mass spectrometry. *Analytical Chemistry*.

[38] Morisseau C, Inceoglu B, Schmelzer K (2010). Naturally occurring monoepoxides of eicosapentaenoic acid and docosahexaenoic acid are bioactive antihyperalgesic lipids. *Journal of Lipid Research*.

[39] Moghaddam MF, Grant DF, Cheek J, Greene JF, Williamson KC, Hammock BD (1997). Bioactivation of leukotoxins to their toxic diols by epoxide hydrolase. *Nature Medicine*.

[40] Xu D, Li N, He Y (2006). Prevention and reversal of cardiac hypertrophy by soluble epoxide hydrolase inhibitors. *Proceedings of the National Academy of Sciences of the United States of America*.

[41] Schmelzer KR, Kubala L, Newman JW, Kim I-H, Eiserich JP, Hammock BD (2005). Soluble epoxide hydrolase is a therapeutic target for acute inflammation. *Proceedings of the National Academy of Sciences of the United States of America*.

[42] Liu JY, Yang J, Inceoglu B (2010). Inhibition of soluble epoxide hydrolase enhances the anti-inflammatory effects of aspirin and 5-lipoxygenase activation protein inhibitor in a murine model. *Biochemical Pharmacology*.

